# Visualization and Analysis of Hepatitis C Virus Structural Proteins at Lipid Droplets by Super-Resolution Microscopy

**DOI:** 10.1371/journal.pone.0102511

**Published:** 2014-07-11

**Authors:** Dennis Eggert, Kathrin Rösch, Rudolph Reimer, Eva Herker

**Affiliations:** 1 Heinrich-Pette-Institute, Leibniz Institute for Experimental Virology, University of Hamburg, Hamburg, Germany; 2 Institute of Physical Chemistry, University of Hamburg, Hamburg, Germany; University of Texas Medical Branch at Galveston, United States of America

## Abstract

Cytosolic lipid droplets are central organelles in the Hepatitis C Virus (HCV) life cycle. The viral capsid protein core localizes to lipid droplets and initiates the production of viral particles at lipid droplet–associated ER membranes. Core is thought to encapsidate newly synthesized viral RNA and, through interaction with the two envelope proteins E1 and E2, bud into the ER lumen. Here, we visualized the spatial distribution of HCV structural proteins core and E2 in vicinity of small lipid droplets by three-color 3D super-resolution microscopy. We observed and analyzed small areas of colocalization between the two structural proteins in HCV-infected cells with a diameter of approximately 100 nm that might represent putative viral assembly sites.

## Introduction

Hepatitis C virus (HCV) persistently infects ∼180 million people worldwide and the associated morbidity and mortality are a major public health concern [1]. Vaccines are not available and albeit recent advances in therapy through development of direct-acting antivirals, current treatment regimens remain very challenging. HCV is a single positive-stranded RNA virus of the *Flaviviridae* family that is translated into a single polyprotein upon entry into host cells. Host and viral proteases cleave this polyprotein, releasing the 10 individual viral proteins. The structural proteins, the viral capsid core and the envelope glycoproteins E1 and E2, are the components of virions while the nonstructural proteins NS3–5B form the viral RNA replication complex. The establishment of fully permissive cell culture systems (HCVcc) [2], [3] revealed a close connection between host cell lipids and HCV replication at each step of the viral replication cycle reviewed in [4]. Interestingly, the cellular storage organelles of lipids, lipid droplets, emerged as putative viral assembly sites [5]–[7].

Two viral proteins localize to lipid droplets in the absence of full viral replication: the capsid protein core and the non-structural protein NS5A [8], [9]. Other viral proteins are found in close-proximity of lipid droplets in infected cells, but they lack intrinsic lipid droplet targeting features as they fail to localize to lipid droplets in uninfected cells [10]. Interestingly, both core and NS5A require triglyceride biosynthesis for trafficking to lipid droplets as inhibitors of diacylglycerol acyltransferase-1 (DGAT1) impair their lipid droplet localization [7], [10].

During translation the capsid protein core is released from the polyprotein by two subsequent cleavages, which generate a 179-amino acid mature protein that is believed to migrate via lateral diffusion into ER subcompartments, to mitochondria and onto the surface of lipid droplets [11]–[13]. Core binds to lipid droplets via an amphipathic helix turn helix motif and mutations that prevent trafficking of the core protein to lipid droplets strongly inhibit virus assembly [5], [6], [14]. During virus assembly, the core protein must be retrieved from the surface of lipid droplets to the site of viral budding at the opposing ER membrane and interaction between viral NS2 and NS3-4A is essential for this recruitment process [15]. Colocalization of the envelope proteins with the capsid core, the prerequisite for assembly events, were analyzed by confocal laser scanning microscopy and either not detected [16], abundant [17], or limited to areas adjacent to small lipid droplets [15]. However, visualizing the HCV assembly process was largely unsuccessful so far as confocal laser scanning microscopy lacks the resolution required and electron microscopic analysis is hindered by the rarity of HCV assembly processes and the heterogeneity of the viral particles [18].

The major drawback of fluorescence microscopy is its limited resolution. The resolution of a microscope is limited by the diffraction barrier to approximately half of the wavelength of the emitted or diffracted light [19]. In three-dimensional fluorescence microscopy like confocal laser scanning microscopy the axial resolution is even worse than the lateral, resulting in a maximal resolution of 200 nm laterally and 500 nm axially for blue emission light [20]. In recent years several microscopy techniques have been developed that overcome the diffraction limit. The most prominent approaches are stimulated emission depletion STED [21], structured illumination microscopy SIM [22], (fluorescence) photoactivated localization microscopy (f)PALM [23], [24], and (direct) stochastic optical reconstruction microscopy (d)STORM [25], [26]. The latter, (f)PLAM, STORM, and dSTORM, have in common that their resolution improvement is based on the precise localization of single fluorescent molecules, therefore they are sometimes summed up as single molecule localization microscopy or in short localization microscopy. The maximum resolution in localization microscopy (with commercially available microscopes) is 20 nm laterally and 50 nm axially [27], a tenfold resolution improvement in all three dimensions compared to confocal laser scanning microscopy. Therefore, the volume in which colocalization is detected is up to 1000× smaller in localization microscopy than in confocal microscopy resulting in much more precise colocalization analyses. The improvement in colocalozation precision has recently been shown in 2D [28]. Localization microscopy has been used successfully to get new insights in structural details or infection processes of pathogens like bacteria [29], [30], plant infecting fungi [31], and human pathogenic viruses like HIV [32]–[34].

Here, for the first time simultaneous three-color 3D dSTORM is employed to study a viral infection. We visualize and analyze the spatial distribution of HCV structural proteins core and E2 in vicinity of lipid droplets, the putative viral assembly site.

## Results

### Lipid droplets can be visualized in dSTORM

**Figure 1:**
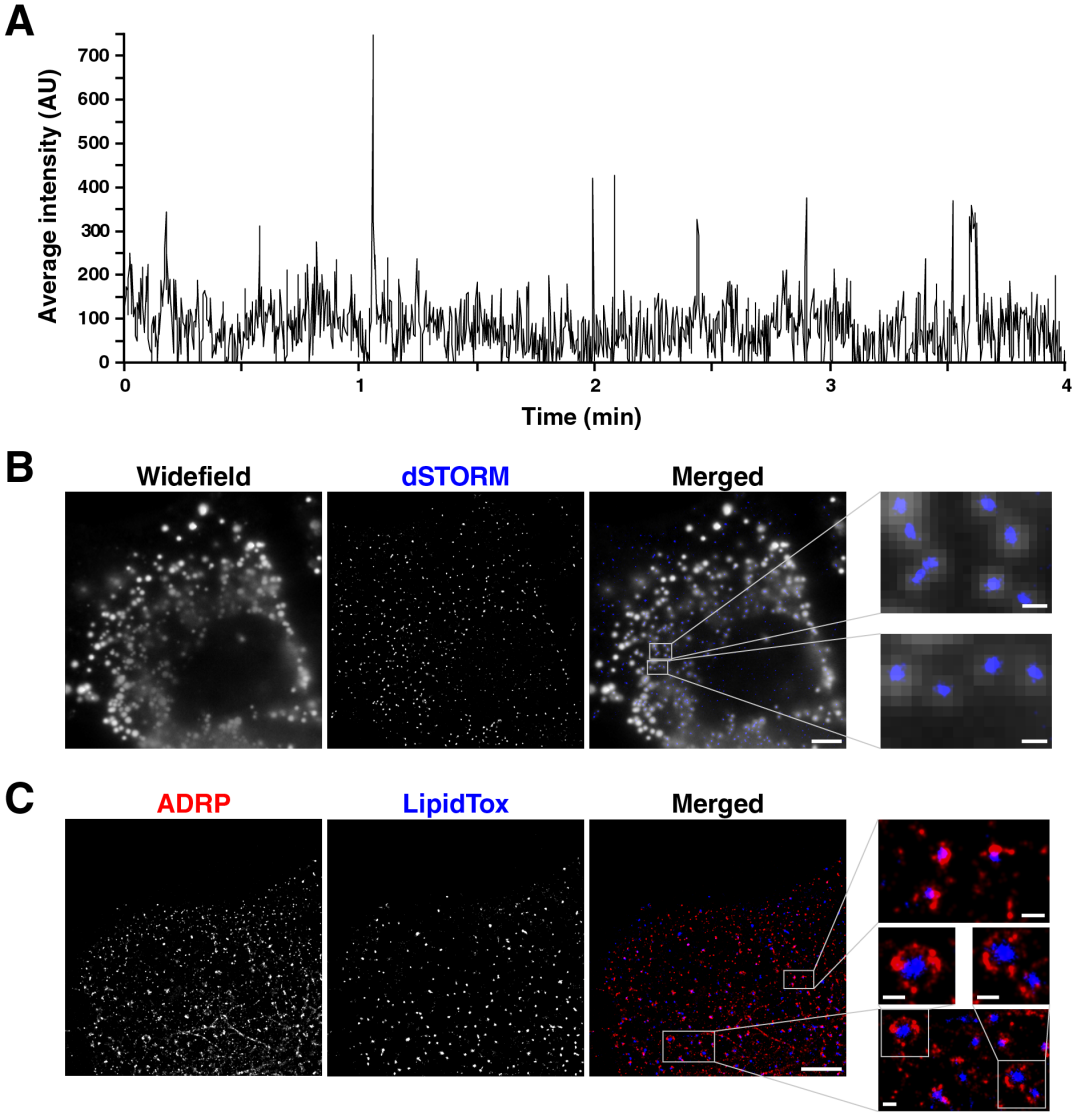
Visualization of lipid droplets in dSTORM. (**A**) Huh7 Lunet cells were incubated with LipidTox Red neutral lipid stain and analyzed by dSTORM. Time-dependent switch between “ON” and “OFF” states of LipidTox Red, shown is the signal of a dSTORM time series. (**B**) Huh7 Lunet cells were incubated with LipidTox Red neutral lipid stain and analyzed by widefield microscopy and dSTORM. Single channels are shown in black and white; the merged image is pseudocolored with widefield in white and dSTORM in blue (scale bar 5 µm, in magnified images 0.5 µm). (**C**) dSTORM of Huh7 Lunet cells after immunostaining with antibodies directed against endogenous ADRP/perilipin 2 and staining of lipid droplets with LipidTox red. Single channels are shown in black and white; the merged image is pseudocolored with ADRP in red and LipidTox in blue (scale bar 5 µm, in magnified images 0.5 µm).

To visualize lipid droplets and surrounding structures by super-resolution microscopy (dSTORM) we first analyzed different lipophilic fluorescent dyes (BODIPY, LipidTox Green, LipidTox Red) for their ability to blink upon excitation. Of the dyes tested, LipidTox Red showed the best properties for dSTORM on our microscope. We validated the ON and OFF switch of LipidTox Red signal over time. Shown is the signal intensity of the fluorophore over several minutes (Figure 1A). To verify that the signals detected in dSTORM represent lipid droplets we correlated widefield fluorescence microscopy of LipidTox Red stained Huh7 Lunet cells with the corresponding dSTORM image. The signals detected with each method overlapped almost completely, with the dSTORM signal usually fitting in the middle of larger signals detected by widefield microscopy (Figure 1B). The lipid droplets we observe in dSTORM mode are generally small (<0.5 µm in diameter) as they are only detectable up to 2 µm axial distance from the coverslip. The large lipid droplets additionally observed in widefield images are mainly more than 2 µm above the coverslip (Figure S1), therefore of those large lipid droplets we only detect the edge in dSTORM mode (Figure S1, arrows). Lipid droplets in hepatoma cells can also be detected by staining with antibodies directed against adipose differentiation-related protein (ADRP)/perilipin 2, that is strongly expressed in liver cells and associates with lipid droplets. In two-dimensional dSTORM images ADRP signals either overlapped with lipid droplets stained with LipidTox Red or tightly surrounded them (Figure 1C, inlays). Therefore, LipidTox Red is suitable for staining lipid droplets in dSTORM experiments.

### Construction and validation of JFH1^Flag-E2^ and Jc1^Flag-E2^ infectious HCV clones

**Figure 2:**
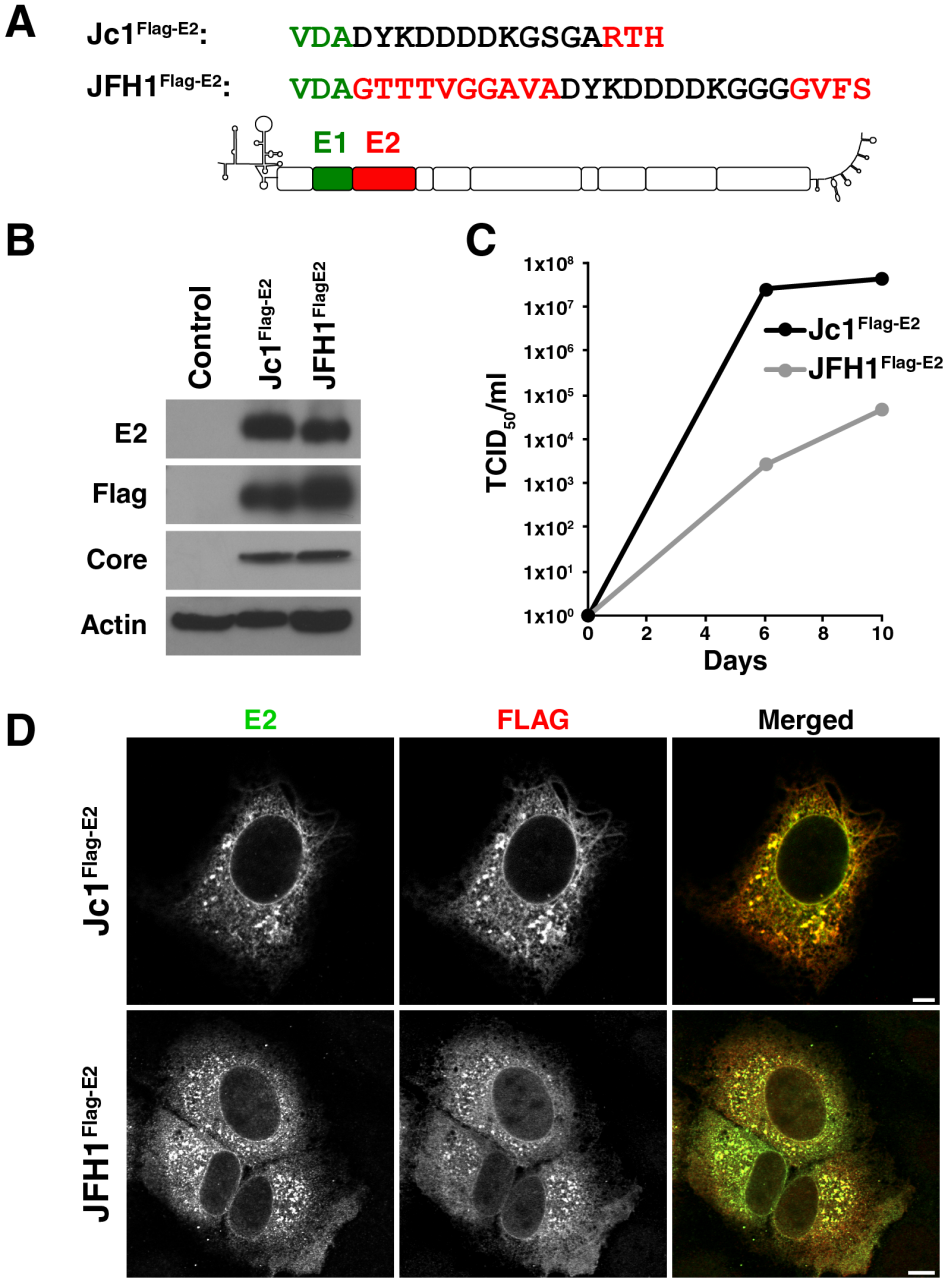
Characterization of Jc1^Flag-E2^ and JFH1^Flag-E2^ infectious HCVcc constructs. (**A**) Scheme of the JFH1^Flag-E2^ and Jc1^Flag-E2^ constructs used in this study. (**B**) Western blot analysis of Huh7.5 cells transfected with *in vitro* transcribed JFH1^Flag-E2^ and Jc1^Flag-E2^ RNA with anti-Flag, anti-E2, anti-core, anti-actin antibodies. (**C**) TCID_50_ of Huh7.5 cells transfected with *in vitro* transcribed JFH1^Flag-E2^ and Jc1^Flag-E2^ RNA. (**D**) Confocal microscopy of Huh7 Lunet cells infected with JFH1^Flag-E2^ and Jc1^Flag-E2^ viral stocks after immunostaining with antibodies recognizing E2 and Flag. Single channels are shown in black and white; the merged image is pseudocolored with ADRP in red and LipidTox in blue (scale bars 5 µm).

To study the subcellular localization of core and E2, we constructed Flag-E2-tagged variants of two infectious HCV clones, the original isolate that replicates in cultured hepatoma cells (JFH1), and a genotype 2a/2a chimera (Jc1) that produces high titers of infectious particles (Figure 2A). To produce infectious HCVcc particles, Huh7.5 cells were transfected with *in vitro* transcribed viral RNA and expression of the viral proteins core and Flag-E2 was verified by western blotting (Figure 2B). Transfected cells produced progeny particles with infectious titers reaching up to 4×10^4^ TCID_50_/ml for JFH1^Flag-E2^- and 4×10^7^ TCID_50_/ml for Jc1^Flag-E2^-infected Huh7.5 cells (Figure 2C). Culture supernatant was then used to infect Huh7 Lunet cells, a subtype of Huh7 cells that is highly permissive to HCV infection and has a superior flat morphology than Huh7.5 cells for the subsequent microscopy experiments [35]. To verify the suitability of anti-Flag staining to localize Flag-E2 we performed co-immunostaining with Flag and E2 antibodies. Signals for Flag and E2 overlapped almost completely (Pearson Coefficient *r_P_* (Jc1^Flag-E2^) = 0.92; *r_P_* (JFH1^Flag-E2^) = 0.852), indicating the suitability of the Flag antibodies to localize Flag-E2 protein (Figure 2D).

### Confocal microscopy of JFH1^Flag-E2^- and Jc1^Flag-E2^-infected cells

Infected cells were then processed for immunostaining with antibodies directed against Flag (E2) and core. Previously it has been suggested that different permeabilization methods might influence lipid droplet localization of select lipid droplet binding proteins [36]. Therefore, we compared two different permeabilization methods, 5 min 0.1% Triton X-100 vs. 5 min 0.5% saponin and addition of 0.1% saponin to the antibody staining solutions. Following the incubation with primary and secondary antibodies, cells were stained with LipidTox Red to visualize lipid droplets and analyzed by confocal microscopy. Signal intensities of core were stronger in JFH1^Flag-E2^- than in Jc1^Flag-E2^-infected cells with core partially localizing to lipid droplets Jc1^Flag-E2^-infected cells and tightly surrounding larger lipid droplets in JFH1^Flag-E2^-infected cells (figure S2). Flag-E2 displayed a predominantly reticular localization and partial colocalization with core at lipid droplets. We measured the degree of colocalization between the two viral proteins according to Manders. The Manders overlap coefficient indicates the portion of the intensity in each channel that overlaps with some intensity in the other channel and is accordingly calculated for each fluorophore. Flag-E2 partially colocalized with core (M1(Jc1^Flag-E2^) = 0.31±0.15, M1(JFH1^Flag-E2^) = 0.45±0.14), while most of core colocalized with Flag-E2 (M2(Jc1^Flag-E2^) = 0.97±0.04, M2(JFH1^Flag-E2^) = 0.87±0.17). We did not observe statistically significant differences in colocalization of core with Flag-E2 between the two viral strains. In our hands both permeabilization methods yielded similar results in confocal microscopy, although permeabilization with Triton X-100 resulted in stronger fluorescence signals than permeabilization with saponin (figure S2).

### Super-resolution microscopy of Jc1^Flag-E2^- and JFH1^Flag-E2^-infected cells

**Figure 3:**
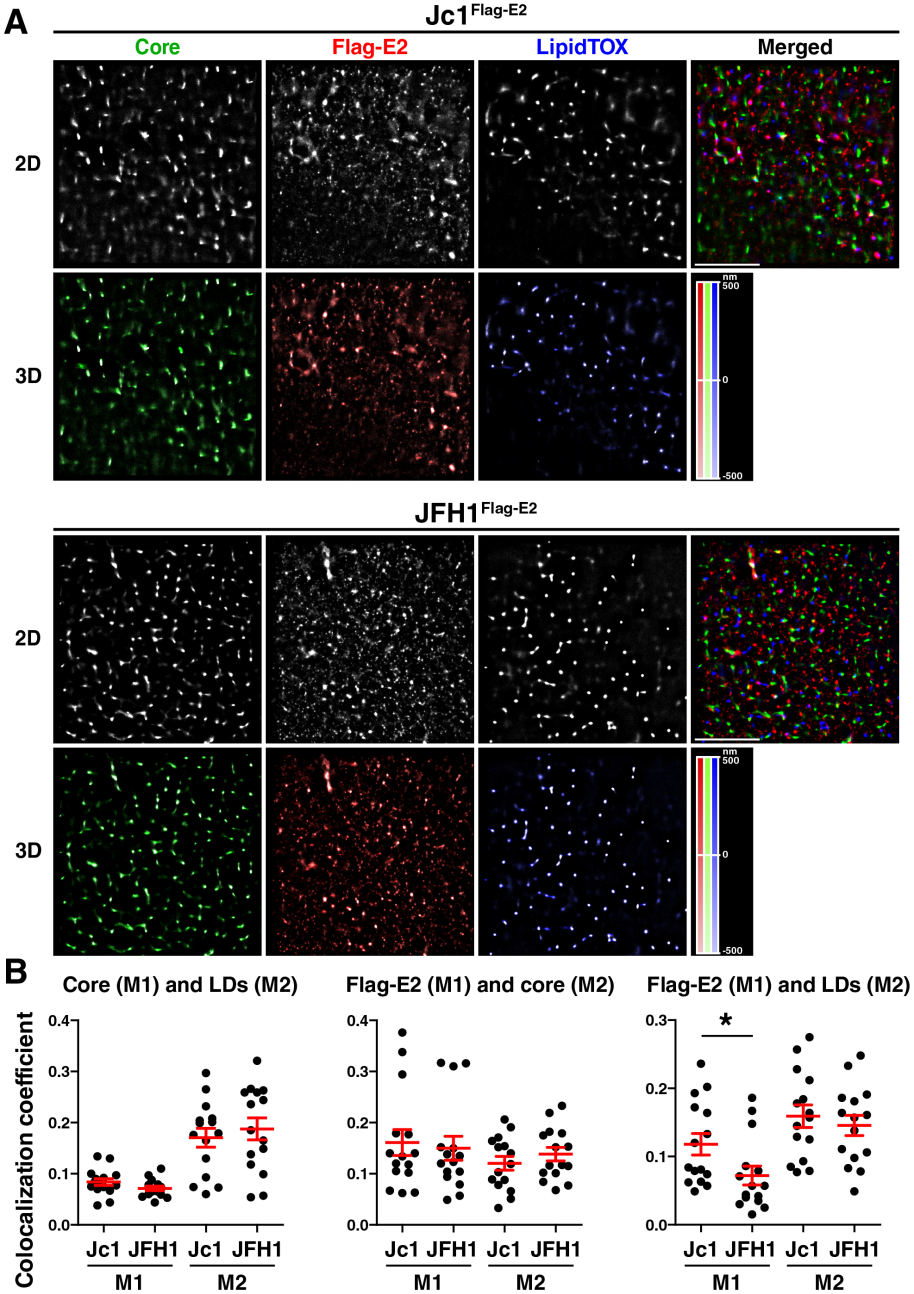
2D and 3D dSTORM images of core, Flag-E2, and lipid droplets in JFH1^Flag-E2^- and Jc1^Flag-E2^-infected cells. Huh7 Lunet cells were infected with JFH1^Flag-E2^ and Jc1^Flag-E2^ viral stocks and processed for immunofluorescence staining. Cells were permeabilized with Triton X-100 followed by staining with anti-core and anti-Flag antibodies and LipidTox Red. 3D super-resolution datasets were acquired using astigmatism imaging. dSTORM images were reconstructed from a time series of 10,000–15,000 raw images per channel (A) Shown are the single channel 2D projections and the merge and 3D images with the axial position color-coded according to the scale on the right. Scale bars of the x–y image represent 5 µm. (B) dSTORM datasets were analyzed for the degree of colocalization using the JACoP (Just Another Colocalization Plugin) plugin for Image J [42]. We analyzed cytosolic regions of interest in 2D of 4 independent experiments and calculated the degree of colocalization using the Manders colocalization coefficient (mean ± sem, *p<0.05, unpaired two-tailed student's t-test).

Super-resolution datasets were then acquired on a custom modified Nikon N-STORM microscope in dSTORM mode [26]. For 3D dSTORM we used astigmatism imaging as described [27]. Super-resolution images were reconstructed from a series of 10,000–15,000 widefield images per channel using the N-STORM v. 2.0 module of NIS Elements AR v. 4.0. (Nikon). We next defined cytosolic regions of interests (ROIs) that were reconstructed in 3D with the axial position information encoded according to the color-coded bar. Shown are example ROIs of 3 independent experiments with Triton X-100–permeabilized and one experiment with saponin-permeabilized cells of Huh7 Lunet cells infected either with Jc1^Flag-E2^ or JFH1^Flag-E2^ (Figure 3 and Figures S3, S4). For each ROI the projected 2D image and the merge as well as the color-coded 3D image are shown. Core staining was generally confined to more distinct areas than the Flag-E2 staining (Figure 3 and Figures S3, S4). Lipid droplets appeared as tight round spots when located close to the coverslip but displayed more scattered signal distribution when further apart from the coverslip, which usually represents the edge of larger droplets (Figure S1). When we compared the two viral strains or the two different permeabilization methods we could not detect any obvious differences. To determine if there are differences in the degree of colocalization of the viral proteins between the two viral strains we calculated the degree of colocalization according to Manders (measuring the portion of the intensity in each channel that coincides with some intensity in the other channel). Overall colocalization coefficients were very low reflecting the high resolution of the dSTORM images. While the levels of colocalization of core with lipid droplets and colocalization of Flag-E2 with core were similar in both viral strains, Flag-E2 displayed slightly less colocalization with lipid droplets in JFH1^Flag-E2^- versus Jc1^Flag-E2^-infected cells, indicating more efficient recruitment of Flag-E2 protein to lipid droplets in cells infected with a viral strain that produces higher titers of progeny virions (Figure 3B).

### Analysis of the 3D distribution and colocalization of core and Flag-E2 at lipid droplets

**Figure 4:**
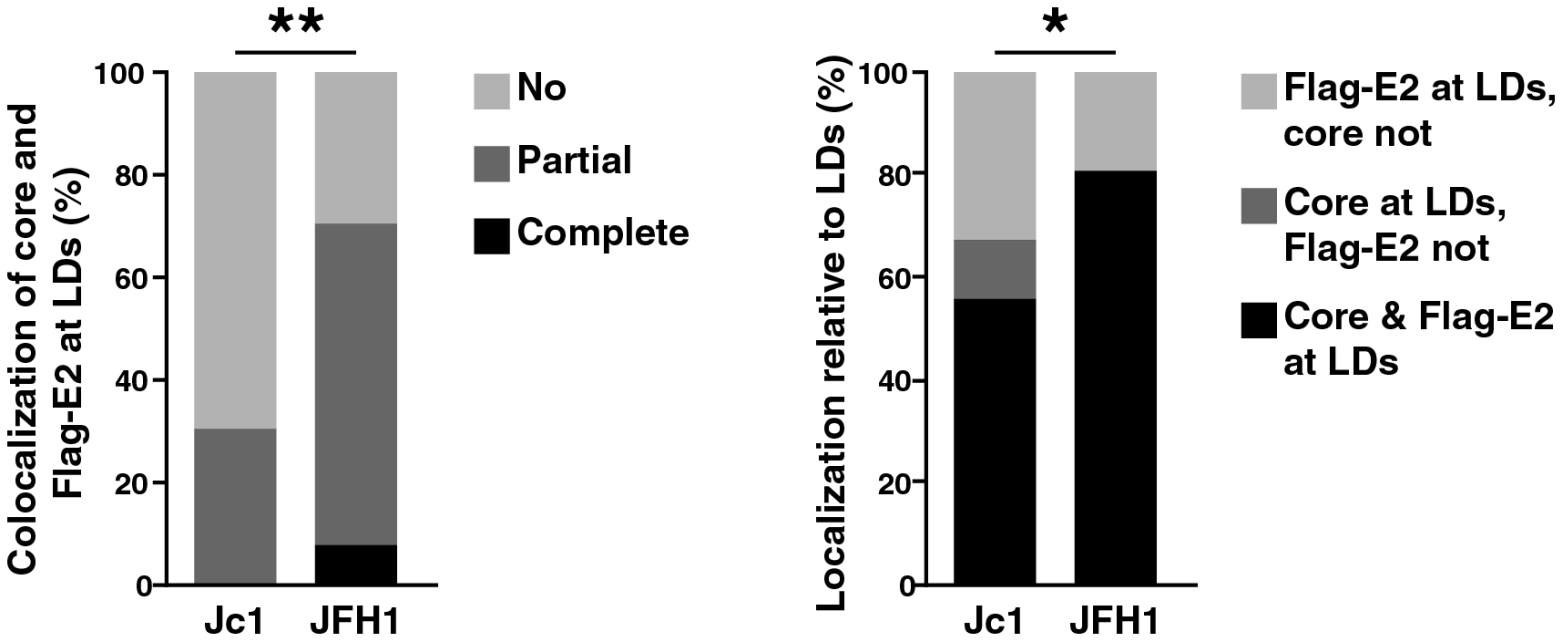
Analysis of the 3D distribution and colocalization of the viral proteins at lipid droplets. dSTORM datasets were analyzed for the distribution and colocalization of core and Flag-E2. Cytosolic regions of interest were reconstructed in 3D and lipid droplets with core and Flag-E2 staining in close proximity were analyzed as follows: lipid droplet localization was scored A) core and Flag-E2 adjacent to lipid droplets, B) Flag-E2 adjacent to core, core adjacent to lipid droplets, and C) core adjacent to Flag-E2, Flag-E2 adjacent to lipid droplets. The degree of colocalization between the viral proteins was scored as I) complete, II) partial, or III) no colocalization. We analyzed reconstructed images from three independent experiments of Triton X-100–permeabilized cells and one experiment of saponin-permeabilized cells with a total amount of 40 lipid droplets in Jc1^Flag-E2^-infected cells and 36 lipid droplets in JFH1^Flag-E2^-infected cells. Colocalization and localization relative to lipid droplets was significantly different between cells infected with the two viral strains (*p<0.05, **p<0.01, chi-square test).

We then analyzed the areas surrounding lipid droplets in regard to the localization of core and Flag-E2 in 3D. Of note, we only analyzed lipid droplets where both viral proteins were detected in close proximity. Lipid droplet localization was scored as follows: A) core and Flag-E2 adjacent to lipid droplets, B) Flag-E2 adjacent to core, core adjacent to lipid droplets, and C) core adjacent to Flag-E2, Flag-E2 adjacent to lipid droplets. Furthermore, the degree of colocalization between the viral proteins was scored as I) complete, II) partial, or III) no colocalization. Analysis of the colocalization between core and Flag-E2 in the area surrounding lipid droplets revealed that in Jc1^Flag-E2^-infected cells there is only at around 30% of the lipid droplets a partial colocalization. In contrast, in JFH1^Flag-E2^-infected cells approximately 70% of the lipid droplets showed colocalization, with ∼10% of lipid droplet surrounding areas showing complete colocalization between core and Flag-E2 (Figure 4). Additionally, in both Jc1^Flag-E2^- and JFH1^Flag-E2^-infected cells core was adjacent to lipid droplets in 70–80% of the analyzed lipid droplets, with Flag-E2 co-coating lipid droplets in 50% (Jc1^Flag-E2^) and 80% (JFH1^Flag-E2^) of the cases (Figure 4). So even though more Flag-E2 is recruited to the sites of viral assembly (Figure 3B), when analyzing individual lipid droplets in 3D less lipid droplets are surrounded by colocalizing core and Flag-E2 proteins.

### Three-dimensional reconstruction of core and Flag-E2 localization in areas surrounding lipid droplets

**Figure 5:**
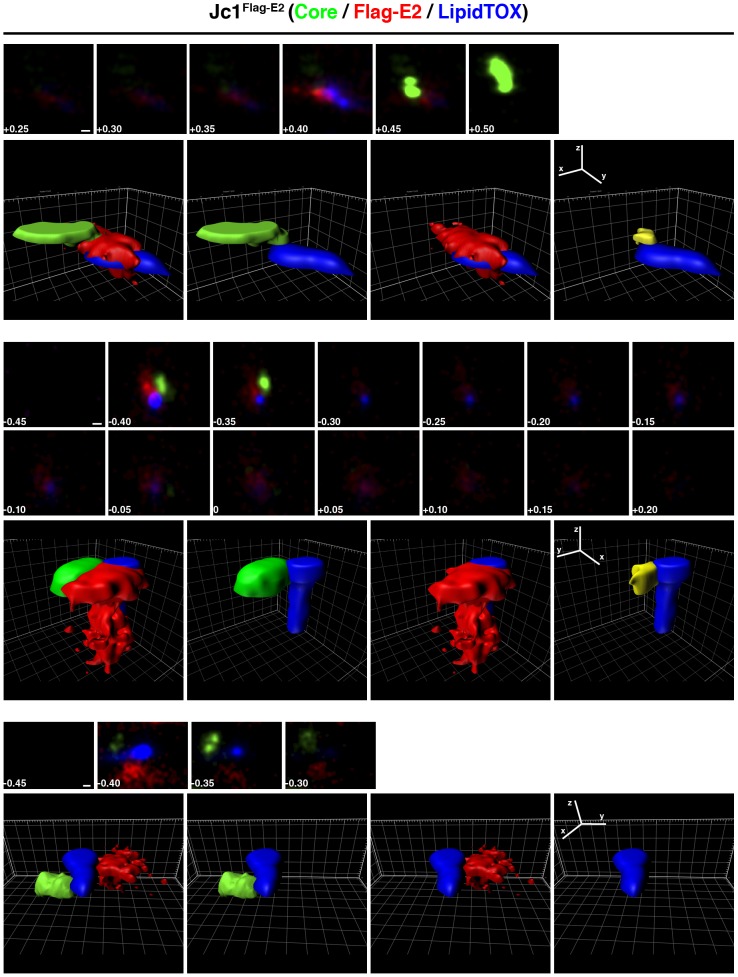
3D reconstitution of core, Flag-E2, and lipid droplets in Jc1^Flag-E2^-infected cells. Representative 3D reconstructions of core and Flag-E2 spatial localization and colocalization around lipid droplets. Shown are three examples of Jc1^Flag-E2^ -infected cells, first the image stacks used for the 3D reconstruction and the 3D reconstruction of all three channels, then core or Flag-E2 and the lipid droplet volume and finally volume of colocalization between core and Flag-E2 and the lipid droplet volume. Scale bars on the image stacks and ruler hatch marks in the 3D images are 100 nm.

**Figure 6:**
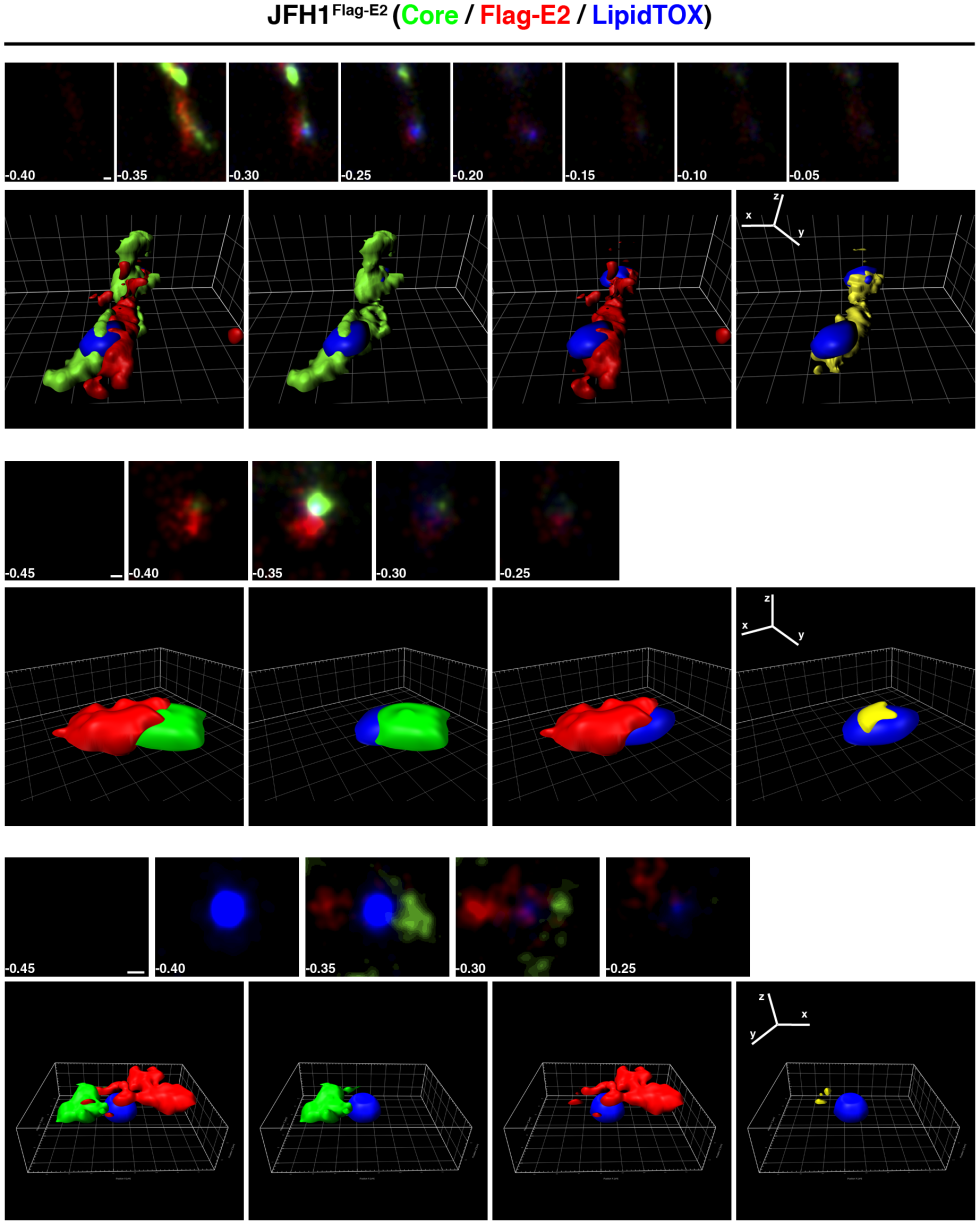
3D reconstitution of core, Flag-E2, and lipid droplets in JFH1^Flag-E2^-infected cells. Representative 3D reconstructions of core and Flag-E2 spatial localization and colocalization around lipid droplets. Shown are three examples of JFH1^Flag-E2^-infected cells, first the image stacks used for the 3D reconstruction and the 3D reconstruction of all three channels, then core or Flag-E2 and the lipid droplet volume and finally volume of colocalization between core and Flag-E2 and the lipid droplet volume. Scale bars on the image stacks and ruler hatch marks in the 3D images are 100 nm.

Next we visualized the spatial distribution of viral proteins surrounding lipid droplets by calculating the volumes occupied by each protein and of the lipid droplets using Imaris software. Depicted are three examples of Jc1^Flag-E2^- and of JFH1^Flag-E2^-infected cells, each showing the stacks that were used to calculate the localization volumes, then the volumes of all three channels, followed by core or Flag-E2 and the lipid droplet volume and finally the volume of colocalization between core and Flag-E2 (in yellow) and the lipid droplet volume (Figure 5 and 6).

In Jc1^Flag-E2^-infected cells core usually was found in distinct spots adjacent to the lipid droplet volume sometimes partially colocalizing with Flag-E2 or on opposing sites of the lipid droplet (Figure 5). The volumes of colocalization were usually around or below 100 nm in diameter. In contrast, in JFH1^Flag-E2^-infected cells core occupied more space in the areas surrounding lipid droplets and the colocalization volumes with Flag-E2 were larger (Figure 6). Overall Flag-E2 occupied volumes that were more scattered than the ones occupied by core.

## Discussion

In this study we performed simultaneous super-resolution microscopy of two viral proteins (core and E2) together with lipid droplets in 3D. We first identified a lipophilic dye suitable for dSTORM imaging and validated the staining with antibodies directed against a lipid droplet-binding protein. To achieve a photoblinking of the fluorophores that was suited for dSTORM the TIRF angle was adjusted to oblique incidence excitation [37]. In this case a thickness of 1–2 µm of the sample is illuminated with maximum light intensity facilitating the blinking of the fluorophores. We observed that within this distance lipid droplets are generally small (0.2–0.5 µm in diameter). In corresponding widefield images larger droplets that are above this area are also visible. Edges of these larger droplets could be detected in dSTORM mode indicated by a scattered staining pattern just below the maximal distance from the coverslip. Well-resolved lipid droplets generally appeared as round to oval shaped objects.

We then analyzed the spatial distribution of core and Flag-E2 of two viral strains (Jc1^Flag-E2^ vs JFH1^Flag-E2^) that differ in the amount of progeny virions they produce. As lipid droplets are the putative assembly sites of HCV we hypothesized to observe distinct differences in the distribution of core and Flag-E2 (as structural proteins they are components of the progeny virions). As noted above we could only analyze rather small lipid droplets, but it has been suggested by confocal microscopic analyses and live cell imaging that these small lipid droplets may represent viral assembly sites [15]. We reconstructed and analyzed the 3D distribution in four independent experiments of cells permeabilized with two different methods. Staining patterns and colocalization events were similar when cells were permeabilized with Triton X-100 or saponin. Over all images acquired, slightly more Flag-E2 colocalizes with lipid droplets in Jc1^Flag-E2^ -infected cells indicating that the recruitment process of the envelope proteins to the putative sites of assembly is more efficient in cells infected with a strain capable of efficient progeny virus production. All other colocalization analyses did not reveal any significant differences.

We next focused our analysis on lipid droplets that had both viral proteins present in close proximity. As noted above colocalization events in projected 2D images were rather infrequent. It has recently been shown that the amount of colocalization decreases in dSTORM compared to confocal microscopy due to the improved resolution [28]. In line with these results, when we analyzed the spatial distribution in 3D only very small areas of colocalization volumes were observed. In addition, the frequency of such core Flag-E2 colocalization events in cells was very low, which could reflect the low number of assembly events in HCV-infected cells. We observed even less colocalization of the capsid protein core with the envelope E2 in cells that produce higher amounts of progeny virions, indicating that assembled virions are transported from the site of assembly or that in the absence of viral assembly, core accumulates and colocalizes with Flag-E2. The volumes of colocalization in Jc1^Flag-E2^-infected cells are generally very tiny, with only around 100 nm in diameter. Within these volumes of colocalization we could not resolve any specific distribution pattern as we are very close to the limit of resolution. In addition, colocalization events were so rare that we could not average the localization of the proteins across colocalization areas of different lipid droplets.

The limitations of our approach are that we can only analyze the space of 2 µm above the coverslip and are therefore unable to analyze the bigger droplets that are, at least in JFH1-infected cells, completely core-coated when analyzed by confocal microscopy. However, the methodology could, combined with correlative electron microscopy, enable finding putative HCV assembly sites in cells by dSTORM, defined through the presence of colocalizing core and Flag-E2 volumes, and thus facilitate analysis of relevant areas in electron microscopy.

## Materials and Methods

### Plasmids

Flag-tagged E2 expressing JFH1 and Jc1 virus constructs were essentially cloned as described [38], [39] by overlap extension PCR using the following primers: BsiWI_J6 sense GGA CAT GAT GAT GAA CTG G, E2_ Flag as CCC TTG TCA TCG TCG TCC TTG TAG TCC GCG TCC ACC CCG GCG GCC, E2_ Flag sense GGA CGA CGA TGA CAA GGG ATC AGG AGC ACG CAC CCA TAC TGT TGG GGG , NS2_JFH1 Not as CCATCGCGGCCGCCGCGCAC and BsiWI_J6 sense GGA CAT GAT GAT GAA CTG G, JFH1 E2_ Flag AS CTT GTC ATC GTC ATC CTT GTA ATC TGC AAC AGC GCC TCC AAC GGT GG , JFH1 E2_ FLAG sense AAG GAT GAC GAT GAC AAG GGA GGC GGT GGC GTG TTC AGC CAT GGC CC , NS2_JFH1 Not as CCA TCG CGG CCG CCG CGC AC (Flag tag underlined).

### Cell lines and culture conditions

Huh7 Lunet cells were a kind gift from Ralf Bartenschlager, Huh7.5 cells were a kind gift from Charles M. Rice. Cells were grown under standard cell culture conditions in high glucose DMEM supplemented with 10% FBS (Biochrom Superior). JFH1^Flag-E2^ and Jc1^Flag-E2^ viral stocks were produced in Huh7.5 cells, while Huh7 Lunet were used for the microscopy studies due to their superior properties for immunofluorescence microscopy [35]. Of note, viral RNA replication and infectious virus release are similar in both cell lines, but Huh7.5 express higher levels CD81 than Huh7 Lunet cells [40].

### Antibodies and reagents

The following antibodies were obtained commercially: anti-core (clone C7-50, Affinity BioReagents), anti-NS5A (clone 2F6/G11, IBT), anti-Flag (F7425, Sigma), anti-ADRP (ab52355, Abcam), anti beta-actin (Sigma), anti-mouse Alexa 488 (Invitrogen), anti-rabbit Alexa 647 (Invitrogen), anti-rabbit-HRP (Jackson Laboratories), anti-mouse-HRP (Jackson Laboratories). Enzymes for molecular cloning were purchased from New England Biolabs, cell culture reagents from Gibco, and fine chemicals, if not noted otherwise, from Sigma. Neutral lipids were stained with HCS LipidTOX Red (H34476, Invitrogen).

### *In vitro* transcription of HCV RNA, production HCV virus stocks, and titer test

HCV viral stocks were prepared as described [7]. Briefly, plasmids encoding JFH1^Flag-E2^ and Jc1^Flag-E2^ viruses were linearized with SspI and purified by phenol-chloroform extraction. *In vitro* transcription was carried out using the MegaScript T7 kit (Ambion). For RNA transfection, Huh7.5 cells were trypsinized, washed once in Opti-MEM (Invitrogen), and resuspended in Cytomix buffer (120 mM KCl, 5 mM MgCl_2_, 0.15 mM CaCl_2_, 2 mM EGTA, 1.9 mM ATP, 4.7 mM GSH, 25 mM HEPES, 10 mM potassium phosphate buffer, pH 7.6) at 10^7^ cells per ml. 400 µl of the cell suspension were mixed with 10 µg HCV RNA and pulsed at 260 V and 950 µF using the Gene Pulser II (Biorad). Culture supernatant of Huh7.5 cells transfected with JFH1^Flag-E2^ and Jc1^Flag-E2^ RNA was harvested, filtered and concentrated by polyethylene glycol 8000 precipitation. For infection experiments, naïve Huh7 Lunet cells were incubated with virus preparations for 3 h at 37°C.

Virus titration was performed by seeding Huh 7.5 cells in 96-well plates and infecting them with serial dilutions of culture supernatants. After 3–4 days, cells were washed with phosphate-buffered saline (PBS), fixed for 10–20 min with methanol at −20°C, permeabilized with 0.1% Triton X-100 and blocked for 1 hour in blocking solution (5% BSA, 1% fish skin gelatin, 50 mM Tris in PBS). Endogenous peroxidase was blocked by a 5 min incubation with PBS containing 0.3% (v/v) hydrogen peroxide. After overnight incubation with anti-NS5A antibodies in blocking solution at 4°C, cells were washed and incubated with anti-rabbit-HRP antibodies for 1 h and subsequently stained with diaminobenzidine (DAB, Roche) substrate for 10 min. Nuclei were counterstained with hematoxylin.

### Western blotting

For western blot analysis cells were lysed in RIPA buffer (1% NP-40, 0.5% sodium deoxycholate, 0.1% SDS in PBS supplemented with protease inhibitor cocktail (Sigma)) and mixed with Laemmli buffer for SDS-PAGE. For chemiluminescent detection we used ECL Lumilight (Roche) and ECL Hyperfilm (Amersham).

### Immunofluorescence and HCS LipidTox Red neutral lipid staining

Cells grown on coverslip or in labtec II chamber slides (Nunc) were fixed in 4% paraformaldehyde for 1 h at room temperature, washed with PBS, and permeabilized in 0.1% Triton X-100 for 5 min or 0.5% saponin for 5 min. After incubation in blocking solution (5% BSA, 1% fish skin gelatin, 50 mM Tris in PBS) for 1 h, cells were incubated with primary antibodies in blocking solution for 1 h, washed and incubated with secondary antibodies for 1 h. When cells were permeabilized with saponin we added 0.1% saponin to the antibody staining solutions. For lipid droplet staining, fixed cells were stained for 20 min with HCS LipidTox Red neutral lipid stain (Invitrogen), diluted 1000-fold in PBS solution. Coverslips were embedded in Mowiol (Calbiochem) mounting medium [41].

### Confocal microscopy

CLSM was either performed on a Nikon C2+ confocal laser scanning microscope or on a LSM 510 (Zeiss) confocal laser scanning microscope. The Nikon C2+ microscope was equipped with four lasers: a 405 nm diode laser (100 mW, Coherent Inc.), a 488 nm DPSS Laser (10 mW, Melles Griot GmbH), a 543 nm HeNe laser (5 mW, Melles Griot GmbH), and a 642 nm diode laser (45 mW, Melles Griot GmbH). The intensity of the laserlight was controlled by an acusto optical tunable filter (AOTF). We used a 60× violet corrected oil objective with a NA of 1.4 for imaging (Plan Apo VC 60× H, Nikon). The LSM 510 microscope was equipped with three lasers: a multiline argon laser (458 nm, 477 nm, 488 nm, 514 nm, 30 mW, LASOS Lasertechnik), a 543 nm HeNe laser (1 mW, LASOS Lasertechnik) and a 633 nm HeNe laser (5 mW, LASOS Lasertechnik). The intensity of the laser light was controlled by an acusto optical tunable filter (AOTF). For imaging a 63× objective with a NA of 1.4 (Plan Apochromat 63×, Zeiss) was used.

### dSTORM and image analysis

Datasets for dSTORM were acquired on a custom modified Nikon N-STORM microscope equipped with an Apo TIRF 100× oil immersion objective with a numerical aperture of 1.49 (Nikon), an electron multiplying charge-coupled device (EMCCD) camera (iXon+ DU-897, Andor Technology) and a quadband filter composed of a quad line beamsplitter (zt405/488/561/640rpc TIRF, Chroma Technology Corporation) and a quad line emission filter (brightline HC 446, 523, 600, 677, Semrock, Inc.).

For excitation of the fluorophores the following lasers were used: for Alexa Fluor 647 a 647 nm continuous wave visible fiber laser (2RU-VFL-P-300-647 MPB Communications Inc.), for Lipid Tox Red a 150 mW 561 nm optically pumped semiconductor laser (Sapphire 561 LP, Coherent, Inc.), and for Alexa Fluor 488 the 488 nm line of an argon gas laser (35-IMA-840-019, Melles Griot GmbH). A 405 nm diode laser (CUBE 405-100C, Coherent Inc.) was used for switching back the fluorophores from the dark to the fluorescent state. For multicolour imaging the lasers were switched on and off alternately controlled by an acoustooptic optical tunable filter (AOTF). The integration time of the EMCCD camera was set to 50–70 ms per frame with an EM gain of 300.

The TIRF angle was adjusted to oblique incidence excitation as described [37]. This allowed super-resolution imaging 1–2 µm deep into the sample. The focus was kept stable during acquisition using Nikon's perfect focus system.

Super-resolution images were reconstructed from a series of 10,000–15,000 images per channel using the N-STORM analysis module v. 2.0.0.76 of NIS Elements AR v. 4.00.07 (Laboratory imaging s.r.o.). 3D super-resolution microscopy was performed using an astigmatism-based approach as described [27]. For each color channel a separate 3D calibration curve was used. 3D super-resolution images were visualized using Bitplane Imaris 7.6.1. software.

The imaging was performed in LabTek II chamber slides using an imaging buffer containing 100 mM β-Mercaptoethanolamin (MEA) as described [37] to facilitate a sufficient blinking of the fluorophores.

## Supporting Information

Figure S1 In dSTORM mode only small lipid droplets can be visualized. Huh7 Lunet cells infected with Jc1^Flag-E2^ viral stocks were incubated with LipidTox Red neutral lipid stain and analyzed by dSTORM and widefield microscopy. Shown are the single channel 2D and 3D images with the axial position color-coded according to the scale on the right (same image as in Figure 3A upper panel) together with the corresponding widefield image. Scale bar represents 5 µm. Arrows highlight the large lipid droplets observed in widefield microscopy. In dSTORM mode only the lowest edge of those large lipid droplets is visible due to oblique incidence illumination.
(TIF)

Figure S2 Confocal microscopy of JFH1^Flag-E2^- and Jc1^Flag-E2^-infected cells. Huh7 Lunet cells were infected with JFH1^Flag-E2^ and Jc1^Flag-E2^ viral stocks and processed for immunofluorescence staining. Cells were either permeabilized with Triton X-100 (A) or saponin (B) followed by staining with anti-core and anti-Flag antibodies and LipidTox Red. Samples were analyzed by confocal microscopy. Single channels are shown in black and white; the merged image is pseudocolored with core in green, Flag-E2 in red, and LipidTox in blue (scale bars 5 µm). Colocalization was determined using the JACoP Image J plugin.
(TIF)

Figure S3 3D dSTORM images of core, Flag-E2, and lipid droplets in Jc1^Flag-E2^-infected cells. Huh7 Lunet cells were infected with Jc1^Flag-E2^ viral stocks and processed for immunofluorescence staining. Cells were permeabilized with Triton X-100 or saponin followed by staining with anti-core and anti-Flag antibodies and LipidTox Red. Super-resolution datasets were acquired as described above. Shown are the single channel 2D and 3D images with the axial position color-coded according to the scale on the right. Scale bars of the x–y image represent 5 µm.
(TIF)

Figure S4 3D dSTORM images of core, Flag-E2, and lipid droplets in JFH1^Flag-E2^-infected cells. Huh7 Lunet cells were infected with JFH1^Flag-^ viral stocks and processed for immunofluorescence staining. Cells were permeabilized with Triton X-100 or saponin followed by staining with anti-core and anti-Flag antibodies and LipidTox Red. Super-resolution datasets were acquired as described above. Shown are the single channel 2D and 3D images with the axial position color-coded according to the scale on the right. Scale bars of the x–y image represent 5 µm.
(TIF)
