# Supplementary figures and images for: Visualization and Analysis of Hepatitis C Virus Structural Proteins at Lipid Droplets by Super-Resolution Microscopy
Source: PLoS One. 2014 Jul 11;9(7):e102511. doi: 10.1371/journal.pone.0102511 (PMC4094509; doi:10.1371/journal.pone.0102511)

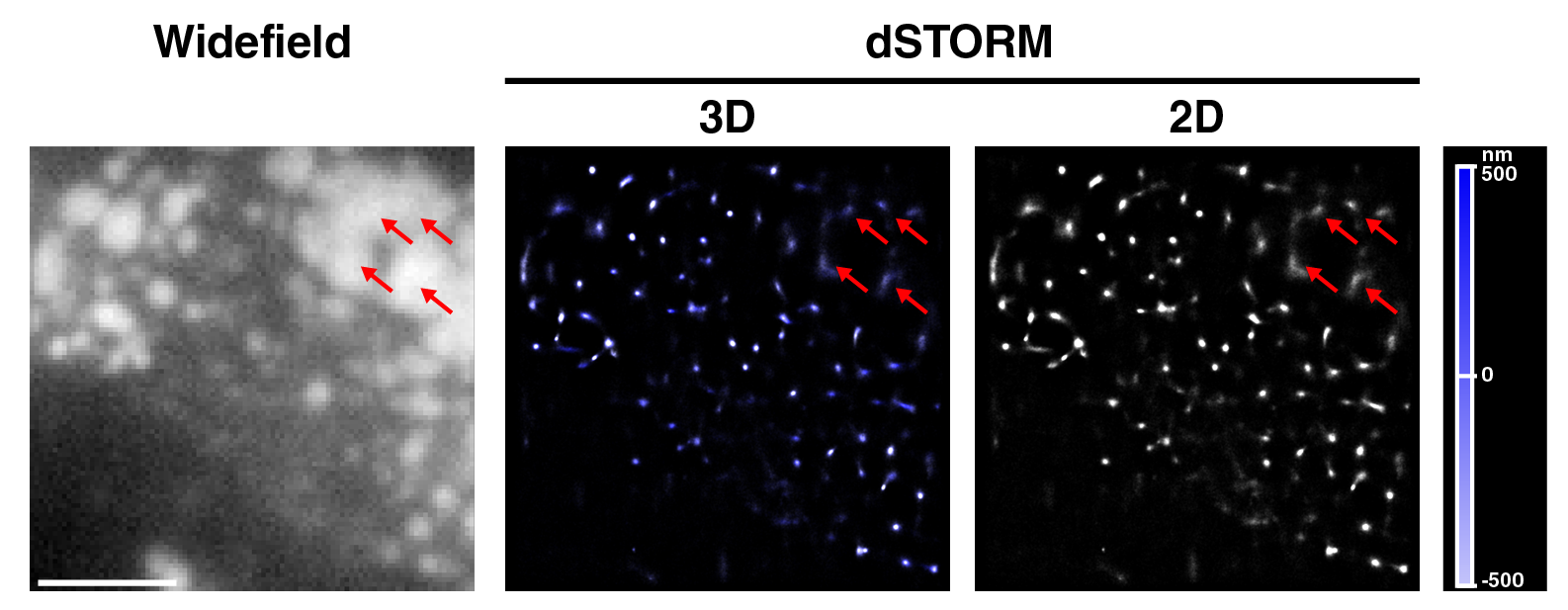

Supplement: Figure S1 — In dSTORM mode only small lipid droplets can be visualized. Huh7 Lunet cells infected with Jc1Flag-E2 viral stocks were incubated with LipidTox Red neutral lipid stain and analyzed by dSTORM and widefield microscopy. Shown are the single channel 2D and 3D images with the axial position color-coded according to the scale on the right (same image as in Figure 3A upper panel) together with the corresponding widefield image. Scale bar represents 5 µm. Arrows highlight the large lipid droplets observed in widefield microscopy. In dSTORM mode only the lowest edge of those large lipid droplets is visible due to oblique incidence illumination. (TIF) [file pone.0102511.s001.tif]

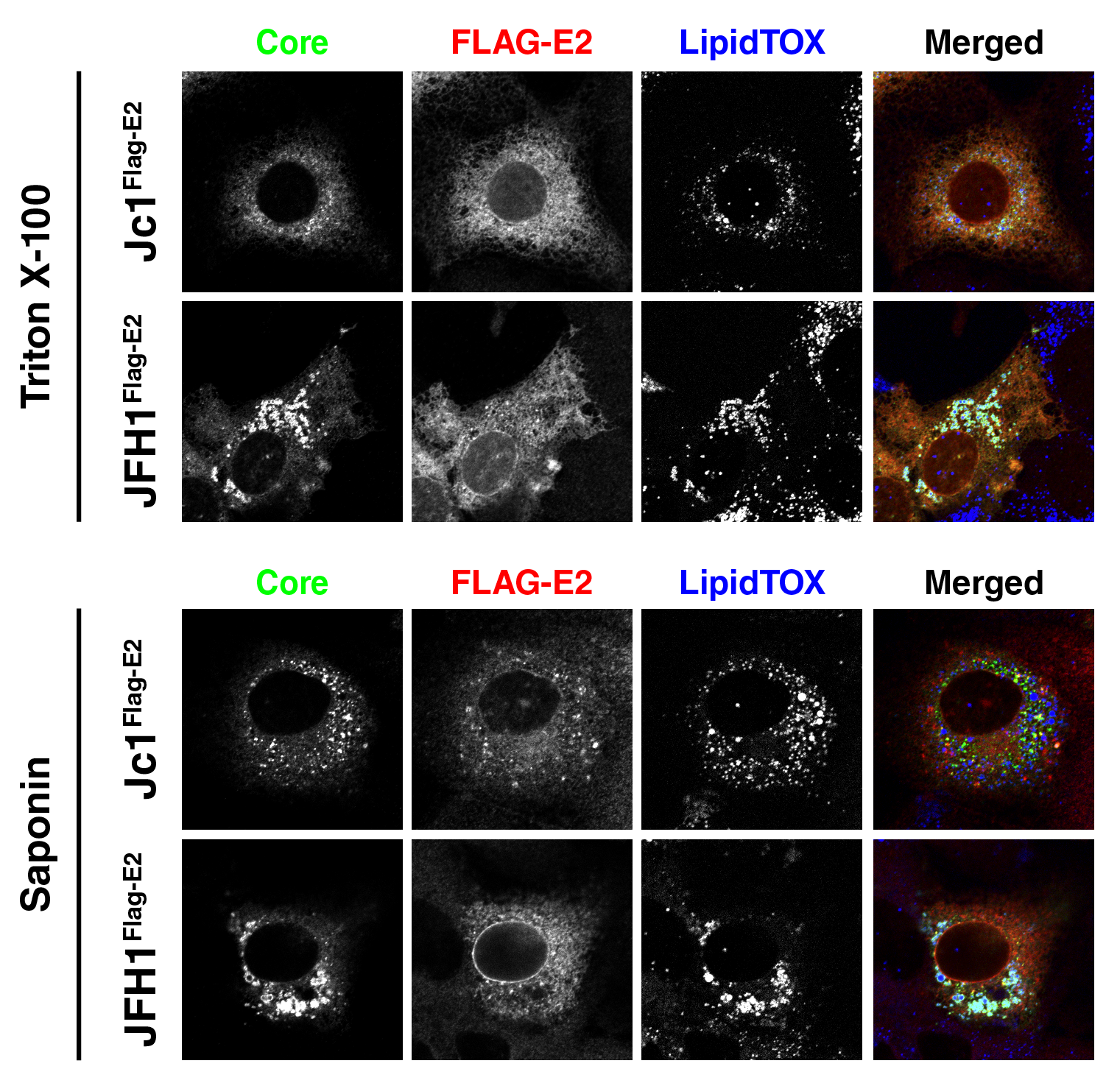

Supplement: Figure S2 — Confocal microscopy of JFH1Flag-E2- and Jc1Flag-E2-infected cells. Huh7 Lunet cells were infected with JFH1Flag-E2 and Jc1Flag-E2 viral stocks and processed for immunofluorescence staining. Cells were either permeabilized with Triton X-100 (A) or saponin (B) followed by staining with anti-core and anti-Flag antibodies and LipidTox Red. Samples were analyzed by confocal microscopy. Single channels are shown in black and white; the merged image is pseudocolored with core in green, Flag-E2 in red, and LipidTox in blue (scale bars 5 µm). Colocalization was determined using the JACoP Image J plugin. (TIF) [file pone.0102511.s002.tif]

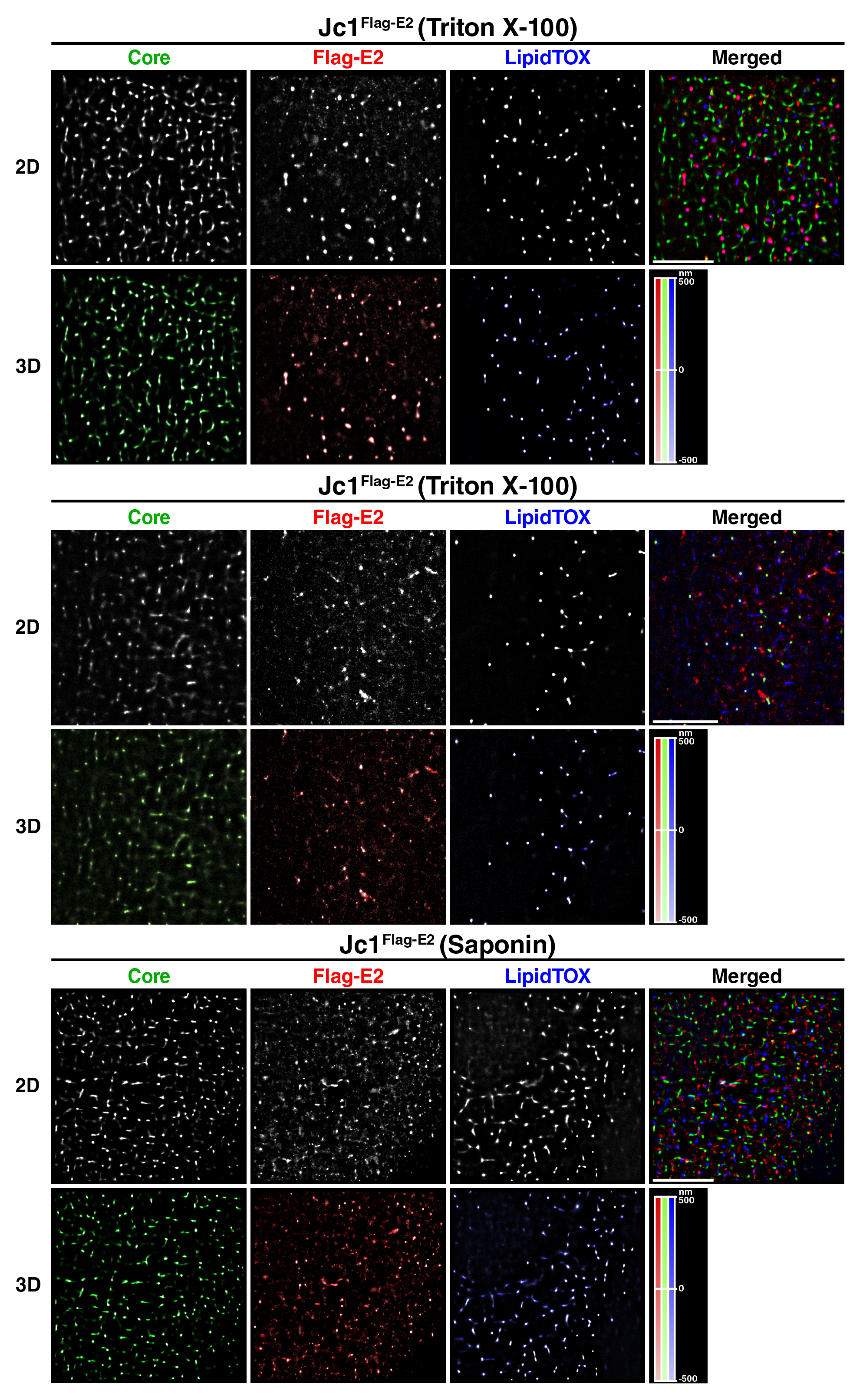

Supplement: Figure S3 — 3D dSTORM images of core, Flag-E2, and lipid droplets in Jc1Flag-E2-infected cells. Huh7 Lunet cells were infected with Jc1Flag-E2 viral stocks and processed for immunofluorescence staining. Cells were permeabilized with Triton X-100 or saponin followed by staining with anti-core and anti-Flag antibodies and LipidTox Red. Super-resolution datasets were acquired as described above. Shown are the single channel 2D and 3D images with the axial position color-coded according to the scale on the right. Scale bars of the x–y image represent 5 µm. (TIF) [file pone.0102511.s003.tif]

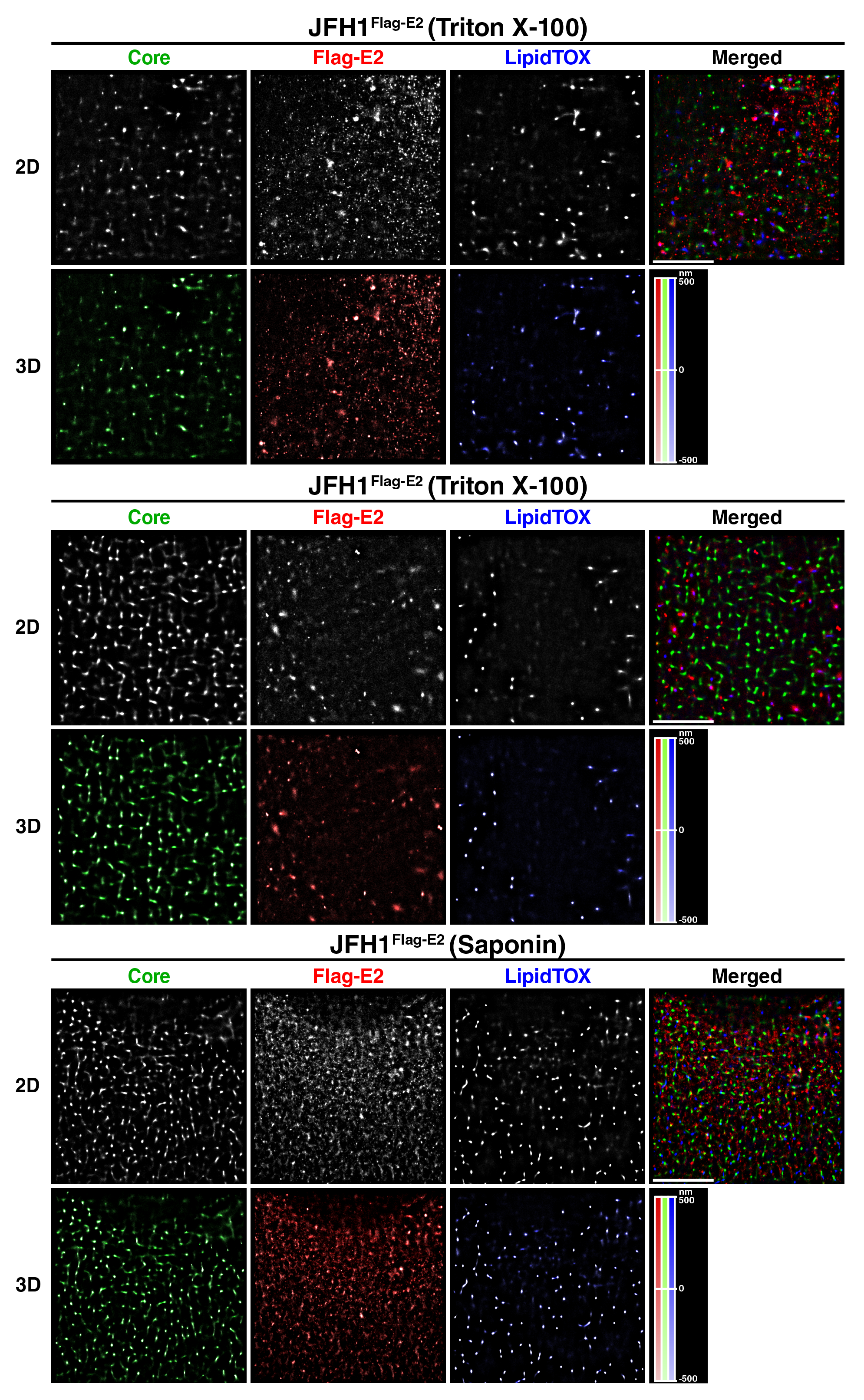

Supplement: Figure S4 — 3D dSTORM images of core, Flag-E2, and lipid droplets in JFH1Flag-E2-infected cells. Huh7 Lunet cells were infected with JFH1Flag- viral stocks and processed for immunofluorescence staining. Cells were permeabilized with Triton X-100 or saponin followed by staining with anti-core and anti-Flag antibodies and LipidTox Red. Super-resolution datasets were acquired as described above. Shown are the single channel 2D and 3D images with the axial position color-coded according to the scale on the right. Scale bars of the x–y image represent 5 µm. (TIF) [file pone.0102511.s004.tif]
